# [μ-1,2-Bis(diphenyl­phosphino)ethane-κ^2^ *P*:*P*′]bis­{[1,2-bis­(diphenyl­phosphino)ethane-κ^2^ *P*,*P*′]cyanidocopper(I)} methanol disolvate

**DOI:** 10.1107/S1600536810029545

**Published:** 2010-07-31

**Authors:** Rong Wang, Ye-Lan Xiao, Qiong-Hua Jin, Cun-Lin Zhang

**Affiliations:** a Department of Chemistry, Capital Normal University, Beijing 100048, People’s Republic of China; b Beijing Key Laboratory for Terahertz Spectroscopy and Imaging, Key Laboratory of Terahertz Optoelectronics, Ministry of Education, Capital Normal University, Beijing 100048, People’s Republic of China

## Abstract

The title centrosymmetric complex, [Cu_2_(CN)_2_(C_26_H_24_P_2_)_3_]·2CH_3_OH, consists of two five-membered [Cu(dppe)CN] rings [dppe is 1,2-bis­(diphenyl­phosphino)ethane] bridged by one μ_2_-dppe ligand, and two methanol solvent mol­ecules. The angles around the central metal atom indicate that each Cu^I^ atom is located in the center of a distorted tetra­hedron. The coordination sphere of each Cu^I^ atom is formed by three P atoms from two dppe ligands, and one C atom from the cyanide ligand. The crystal structure is stabilized by O—H⋯N hydrogen bonds, which are formed by the O—H donor group from methanol and the N-atom acceptor from a cyanide ligand.

## Related literature

For related structures, see: Jin *et al.* (2009 ▶); Effendy *et al.* (2006 ▶); Sivasankar *et al.* (2004 ▶); Di Nicola *et al.* (2006 ▶); Saravanabharathi *et al.* (2002 ▶). For general background to the photophysical properties of similar compounds, see: Cingolani *et al.* (2005 ▶); Song *et al.* (2007 ▶).
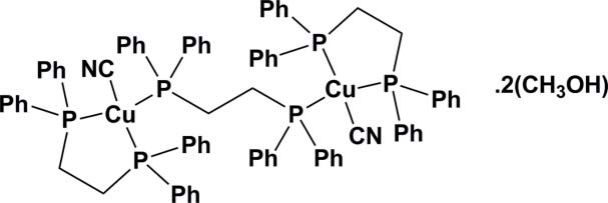


## Experimental

### 

#### Crystal data

[Cu_2_(CN)_2_(C_26_H_24_P_2_)_3_]·2CH_4_O
*M* *_r_* = 1438.38
Monoclinic, 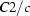

*a* = 23.423 (2) Å
*b* = 17.7912 (16) Å
*c* = 17.6614 (18) Å
β = 92.194 (1)°
*V* = 7354.6 (12) Å^3^
*Z* = 4
Mo *K*α radiation
μ = 0.76 mm^−1^
*T* = 298 K
0.44 × 0.40 × 0.25 mm

#### Data collection

Bruker SMART CCD area-detector diffractometer
Absorption correction: multi-scan (*SADABS*; Sheldrick, 1996 ▶) *T*_min_ = 0.732, *T*_max_ = 0.833
18242 measured reflections
6494 independent reflections
4140 reflections with *I* > 2σ(*I*)
*R*_int_ = 0.044

#### Refinement

*R*[*F* ^2^ > 2σ(*F* ^2^)] = 0.049
*wR*(*F* ^2^) = 0.149
*S* = 1.06
6494 reflections
425 parameters
H-atom parameters constrained
Δρ_max_ = 0.63 e Å^−3^
Δρ_min_ = −0.39 e Å^−3^

### 

Data collection: *SMART* (Bruker, 2007 ▶); cell refinement: *SAINT-Plus* (Bruker, 2007 ▶); data reduction: *SAINT-Plus*; program(s) used to solve structure: *SHELXS97* (Sheldrick, 2008 ▶); program(s) used to refine structure: *SHELXL97* (Sheldrick, 2008 ▶); molecular graphics: *SHELXTL* (Sheldrick, 2008 ▶); software used to prepare material for publication: *SHELXTL*.

## Supplementary Material

Crystal structure: contains datablocks global, I. DOI: 10.1107/S1600536810029545/su2196sup1.cif

Structure factors: contains datablocks I. DOI: 10.1107/S1600536810029545/su2196Isup2.hkl

Additional supplementary materials:  crystallographic information; 3D view; checkCIF report

## Figures and Tables

**Table 1:** Hydrogen-bond geometry (Å, °)

*D*—H⋯*A*	*D*—H	H⋯*A*	*D*⋯*A*	*D*—H⋯*A*
O1—H1⋯N1^i^	0.82	2.02	2.829 (11)	171

Symmetry code: (i) 
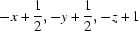
.

**Fig. 1.**
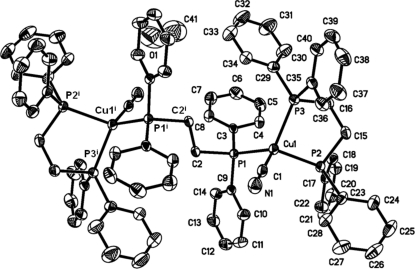
A view of the molecular structure of complex (1), with the displacement ellipsoids drawn at the 50% probability level [Symmetry code: (i) = -x+1/2, -y+1/2, -z+1; Hydrogen atoms have been omitted for clarity].

## supplementary crystallographic information

### Comment

Copper(I) complexes containing the diphosphine ligands bis(diphenylphosphinoethane)(Dppe) are extensively studied because of their interesting structures and photophysical properties (Cingolani *et al.*, 2005; Song *et al.*, 2007). dppe is a very efficient bridging bidentate ligand and its chelating tendency is very suitable to lock the metal atom. As a part of the extension of our study on the systematic structural chemistry of copper(I) complexes with ligands containing phosphine and nitrogen atoms (Jin *et al.*, 2009), we synthesized the new title complex, (1), in the presence of (NH_4_)_2_WS_4_ and 1,10-phenanthroline.

The molecular structure of complex (1) is depicted in Fig. 1. It consists of two five-membered [Cu(dppe)CN] rings that are bridged by one µ_2_-dppe ligand, and two methanol solvent molecules. The copper atom is four-coordinated by three P-atoms from two dppe ligands, and one C-atom from the cyanide ion. The Cu—P distances of 2.2832 (12) Å, 2.3041 (13) Å and 2.3291 (12) Å are longer than those in complex [Cu_2_(dppe)_3_(CN)_2_].2(CH_3_CN) (2), which vary from 2.2784 (4) to 2.3158 (4) Å (Effendy *et al.*, 2006), but are almost equal to those in complex [Cu_2_(dppe)_3_(CN)_2_] (3), which vary from 2.2808 (8) to 2.3276 (8) Å (Saravanabharathi *et al.*, 2002). The Cu—C distance of 1.952 (6)Å in complex (1) is shorter than the same distance observed in complexes (2) and (3); 1.975 (2) Å and 1.964 (4) Å, respectively.

In (1) the P—Cu—C angles are in the range 107.59 (14) - 119.11 (14)°, and the P—Cu—P angles are in the range 89.03 (4) - 115.07 (5)°. This confirms the distored tetrahedral environment around the copper(I) atom. These values are very close to those observed for complex (3), where the P—Cu—C angles range from 107.05 (9) to 120.73 (9)°, and the P—Cu—P angles are in the range 89.22 (3) - 115.16 (3) °.

Though both (NH_4_)_2_WS_4_ and 1,10-phenanthroline were starting materials in the prepartion of (1), they do not appear in the final product. This may be related to the solvent methanol because the O—H donor from methanol can form an O—H···N hydrogen bond with the N atom from the cyanide anion (Table 1), and this can stablize the molecular structure of the complex.

The crystal structure of complex (1) is similar with that of complex (2). Other similar complexes are adducts CuX:dppe:*X*, where *X* is a simple inorganic anion, for example, a halide (Effendy *et al.*, 2006; Di Nicola *et al.*, 2006), thiocyanate (Saravanabharathi *et al.*, 2002), nitrate (Saravanabharathi *et al.*, 2002), perchlorate (Sivasankar *et al.*, 2004; Jin *et al.*, 2009) and tetrafluoroborate (Jin *et al.*, 2009).

### Experimental

A mixture of CuCN, bis(diphenylphosphinoethane), (NH_4_)_2_WS_4_ and 1,10-Phenanthroline, in the molar ratio of 3:3:1:1 in CH_2_Cl_2_ and MeOH (10 ml,V/V=1/1), was stirred for 4 h at RT, then filtered. Subsequent slow evaporation of the filtrate resulted in the formation of yellow crystals of complex (1). Crystals suitable for single-crystal X-ray diffraction were selected directly from the sample as prepared.

### Refinement

The H-atoms were included in calculated positions and treated as riding atoms: O—H = 0.82 Å, C—H 0.93 - 0.96 Å with *U*_iso_(H) = k × *U*_eq_(parent O or C-atom), where k = 1.5 for OH and CH_3_ H-atoms, and k = 1.2 for all other H-atoms.

### Crystal data

**Table:**

[Cu_2_(CN)_2_(C_26_H_24_P_2_)_3_]·2CH_4_O	*F*(000) = 3000
*M**_r_* = 1438.38	*D*_x_ = 1.299 Mg m^−^^3^
Monoclinic, *C*2/*c*	Mo *K*α radiation, λ = 0.71073 Å
Hall symbol: -C 2yc	Cell parameters from 4634 reflections
*a* = 23.423 (2) Å	θ = 2.3–27.3°
*b* = 17.7912 (16) Å	µ = 0.76 mm^−^^1^
*c* = 17.6614 (18) Å	*T* = 298 K
β = 92.194 (1)°	Block, yellow
*V* = 7354.6 (12) Å^3^	0.44 × 0.40 × 0.25 mm
*Z* = 4	

### Data collection

**Table:**

Bruker SMART CCD area-detector diffractometer	6494 independent reflections
Radiation source: fine-focus sealed tube	4140 reflections with *I* > 2σ(*I*)
graphite	*R*_int_ = 0.044
phi and ω scans	θ_max_ = 25.0°, θ_min_ = 1.4°
Absorption correction: multi-scan (*SADABS*; Sheldrick, 1996)	*h* = −23→27
*T*_min_ = 0.732, *T*_max_ = 0.833	*k* = −21→21
18242 measured reflections	*l* = −17→21

### Refinement

**Table:**

Refinement on *F*^2^	Primary atom site location: structure-invariant direct methods
Least-squares matrix: full	Secondary atom site location: difference Fourier map
*R*[*F*^2^ > 2σ(*F*^2^)] = 0.049	Hydrogen site location: inferred from neighbouring sites
*wR*(*F*^2^) = 0.149	H-atom parameters constrained
*S* = 1.06	*w* = 1/[σ^2^(*F*_o_^2^) + (0.063*P*)^2^ + 16.0608*P*] where *P* = (*F*_o_^2^ + 2*F*_c_^2^)/3
6494 reflections	(Δ/σ)_max_ = 0.001
425 parameters	Δρ_max_ = 0.63 e Å^−^^3^
0 restraints	Δρ_min_ = −0.39 e Å^−^^3^

### Special details

**Table:**

Geometry. All e.s.d.'s (except the e.s.d. in the dihedral angle between two l.s. planes) are estimated using the full covariance matrix. The cell e.s.d.'s are taken into account individually in the estimation of e.s.d.'s in distances, angles and torsion angles; correlations between e.s.d.'s in cell parameters are only used when they are defined by crystal symmetry. An approximate (isotropic) treatment of cell e.s.d.'s is used for estimating e.s.d.'s involving l.s. planes.
Refinement. Refinement of *F*^2^ against ALL reflections. The weighted *R*-factor *wR* and goodness of fit *S* are based on *F*^2^, conventional *R*-factors *R* are based on *F*, with *F* set to zero for negative *F*^2^. The threshold expression of *F*^2^ > σ(*F*^2^) is used only for calculating *R*-factors(gt) *etc*. and is not relevant to the choice of reflections for refinement. *R*-factors based on *F*^2^ are statistically about twice as large as those based on *F*, and *R*- factors based on ALL data will be even larger.

### Fractional atomic coordinates and isotropic or equivalent isotropic displacement parameters (Å^2^)

**Table:**

	*x*	*y*	*z*	*U*_iso_*/*U*_eq_	
Cu1	0.26534 (2)	0.24069 (3)	0.29813 (3)	0.03662 (18)	
N1	0.3058 (2)	0.4054 (3)	0.3181 (3)	0.0646 (13)	
O1	0.0834 (4)	0.0342 (6)	0.6565 (6)	0.243 (5)	
H1	0.1155	0.0494	0.6686	0.292*	
P1	0.28874 (4)	0.17964 (6)	0.40872 (6)	0.0305 (3)	
P2	0.30147 (5)	0.18670 (7)	0.18979 (7)	0.0372 (3)	
P3	0.17430 (5)	0.21480 (7)	0.25132 (7)	0.0379 (3)	
C1	0.2892 (2)	0.3452 (3)	0.3102 (3)	0.0437 (11)	
C2	0.28034 (17)	0.2350 (2)	0.4956 (2)	0.0332 (10)	
H2A	0.2907	0.2038	0.5391	0.040*	
H2B	0.3066	0.2771	0.4954	0.040*	
C3	0.25244 (17)	0.0913 (2)	0.4259 (2)	0.0328 (10)	
C4	0.2433 (2)	0.0433 (3)	0.3649 (3)	0.0463 (12)	
H4	0.2558	0.0573	0.3175	0.056*	
C5	0.2161 (2)	−0.0251 (3)	0.3731 (3)	0.0608 (15)	
H5	0.2103	−0.0566	0.3315	0.073*	
C6	0.1977 (2)	−0.0462 (3)	0.4427 (4)	0.0640 (15)	
H6	0.1798	−0.0924	0.4486	0.077*	
C7	0.2057 (2)	0.0011 (3)	0.5039 (3)	0.0594 (14)	
H7	0.1926	−0.0126	0.5510	0.071*	
C8	0.2332 (2)	0.0687 (3)	0.4953 (3)	0.0460 (12)	
H8	0.2389	0.0999	0.5372	0.055*	
C9	0.36466 (17)	0.1560 (2)	0.4211 (2)	0.0359 (10)	
C10	0.4037 (2)	0.1964 (3)	0.3819 (3)	0.0572 (14)	
H10	0.3912	0.2327	0.3472	0.069*	
C11	0.4621 (2)	0.1835 (4)	0.3935 (4)	0.0745 (18)	
H11	0.4882	0.2116	0.3670	0.089*	
C12	0.4810 (2)	0.1305 (4)	0.4432 (3)	0.0676 (17)	
H12	0.5200	0.1215	0.4501	0.081*	
C13	0.4429 (2)	0.0898 (3)	0.4834 (3)	0.0628 (15)	
H13	0.4559	0.0538	0.5181	0.075*	
C14	0.3848 (2)	0.1026 (3)	0.4721 (3)	0.0504 (13)	
H14	0.3590	0.0747	0.4993	0.060*	
C15	0.23651 (19)	0.1723 (3)	0.1290 (3)	0.0456 (12)	
H15A	0.2270	0.2188	0.1028	0.055*	
H15B	0.2441	0.1345	0.0912	0.055*	
C16	0.18567 (19)	0.1475 (3)	0.1747 (3)	0.0438 (11)	
H16A	0.1929	0.0979	0.1958	0.053*	
H16B	0.1516	0.1447	0.1417	0.053*	
C17	0.33868 (19)	0.0963 (3)	0.1854 (2)	0.0412 (11)	
C18	0.3107 (2)	0.0287 (3)	0.1734 (3)	0.0490 (12)	
H18	0.2711	0.0283	0.1672	0.059*	
C19	0.3406 (2)	−0.0385 (3)	0.1706 (3)	0.0584 (14)	
H19	0.3209	−0.0833	0.1623	0.070*	
C20	0.3982 (3)	−0.0392 (3)	0.1797 (3)	0.0651 (16)	
H20	0.4181	−0.0844	0.1777	0.078*	
C21	0.4273 (2)	0.0267 (4)	0.1920 (4)	0.0714 (17)	
H21	0.4670	0.0264	0.1980	0.086*	
C22	0.3977 (2)	0.0938 (3)	0.1953 (3)	0.0593 (14)	
H22	0.4177	0.1382	0.2045	0.071*	
C23	0.3481 (2)	0.2435 (3)	0.1316 (3)	0.0455 (12)	
C24	0.3554 (2)	0.2283 (3)	0.0564 (3)	0.0606 (14)	
H24	0.3343	0.1901	0.0328	0.073*	
C25	0.3935 (3)	0.2691 (4)	0.0156 (4)	0.0784 (19)	
H25	0.3973	0.2592	−0.0357	0.094*	
C26	0.4252 (3)	0.3234 (4)	0.0498 (4)	0.082 (2)	
H26	0.4515	0.3501	0.0222	0.099*	
C27	0.4192 (2)	0.3396 (3)	0.1245 (4)	0.0757 (18)	
H27	0.4411	0.3772	0.1478	0.091*	
C28	0.3801 (2)	0.2995 (3)	0.1655 (3)	0.0556 (14)	
H28	0.3755	0.3106	0.2164	0.067*	
C29	0.11966 (19)	0.1722 (3)	0.3079 (3)	0.0464 (12)	
C30	0.0944 (3)	0.1036 (3)	0.2908 (4)	0.0761 (18)	
H30	0.1061	0.0757	0.2496	0.091*	
C31	0.0515 (3)	0.0769 (4)	0.3358 (5)	0.101 (2)	
H31	0.0338	0.0313	0.3240	0.122*	
C32	0.0350 (3)	0.1166 (4)	0.3970 (4)	0.098 (2)	
H32	0.0069	0.0974	0.4274	0.117*	
C33	0.0592 (3)	0.1842 (4)	0.4141 (4)	0.084 (2)	
H33	0.0473	0.2115	0.4555	0.101*	
C34	0.1015 (2)	0.2122 (3)	0.3699 (3)	0.0597 (14)	
H34	0.1180	0.2584	0.3817	0.072*	
C35	0.13433 (19)	0.2891 (3)	0.2004 (3)	0.0423 (11)	
C36	0.1637 (2)	0.3484 (3)	0.1707 (3)	0.0548 (13)	
H36	0.2032	0.3509	0.1779	0.066*	
C37	0.1351 (3)	0.4045 (3)	0.1302 (3)	0.0707 (17)	
H37	0.1554	0.4440	0.1096	0.085*	
C38	0.0765 (3)	0.4017 (3)	0.1204 (3)	0.0716 (17)	
H38	0.0573	0.4398	0.0940	0.086*	
C39	0.0469 (2)	0.3436 (3)	0.1492 (3)	0.0648 (16)	
H39	0.0073	0.3419	0.1419	0.078*	
C40	0.0750 (2)	0.2864 (3)	0.1895 (3)	0.0530 (13)	
H40	0.0544	0.2467	0.2090	0.064*	
C41	0.0678 (5)	0.0611 (8)	0.5868 (8)	0.200 (6)	
H41A	0.0315	0.0859	0.5890	0.300*	
H41B	0.0648	0.0202	0.5514	0.300*	
H41C	0.0960	0.0961	0.5707	0.300*	

### Atomic displacement parameters (Å^2^)

**Table:**

	*U*^11^	*U*^22^	*U*^33^	*U*^12^	*U*^13^	*U*^23^
Cu1	0.0365 (3)	0.0446 (3)	0.0287 (3)	0.0026 (2)	0.0002 (2)	0.0009 (2)
N1	0.084 (4)	0.054 (3)	0.057 (3)	−0.012 (3)	0.006 (2)	−0.007 (2)
O1	0.204 (9)	0.280 (12)	0.241 (12)	−0.122 (8)	−0.036 (8)	0.110 (9)
P1	0.0297 (6)	0.0359 (6)	0.0260 (6)	0.0033 (5)	0.0006 (4)	−0.0011 (5)
P2	0.0374 (7)	0.0460 (7)	0.0284 (6)	0.0033 (5)	0.0041 (5)	−0.0016 (5)
P3	0.0319 (6)	0.0469 (7)	0.0346 (7)	0.0047 (5)	−0.0016 (5)	−0.0020 (5)
C1	0.045 (3)	0.057 (3)	0.029 (3)	0.005 (2)	0.003 (2)	0.003 (2)
C2	0.036 (2)	0.036 (2)	0.027 (2)	0.0024 (19)	−0.0035 (18)	−0.0043 (18)
C3	0.031 (2)	0.040 (2)	0.028 (2)	0.0052 (18)	−0.0047 (18)	−0.0014 (19)
C4	0.054 (3)	0.051 (3)	0.034 (3)	0.002 (2)	0.001 (2)	0.000 (2)
C5	0.074 (4)	0.052 (3)	0.056 (4)	−0.009 (3)	−0.010 (3)	−0.014 (3)
C6	0.068 (4)	0.049 (3)	0.075 (4)	−0.012 (3)	−0.005 (3)	0.006 (3)
C7	0.073 (4)	0.053 (3)	0.053 (4)	−0.007 (3)	0.009 (3)	0.012 (3)
C8	0.057 (3)	0.044 (3)	0.037 (3)	−0.004 (2)	0.003 (2)	−0.002 (2)
C9	0.031 (2)	0.045 (3)	0.032 (2)	0.006 (2)	0.0013 (19)	−0.008 (2)
C10	0.041 (3)	0.078 (4)	0.053 (3)	0.000 (3)	0.001 (2)	0.009 (3)
C11	0.043 (3)	0.108 (5)	0.073 (4)	−0.015 (3)	0.009 (3)	0.004 (4)
C12	0.036 (3)	0.098 (5)	0.067 (4)	0.012 (3)	−0.005 (3)	−0.013 (4)
C13	0.049 (3)	0.078 (4)	0.060 (4)	0.019 (3)	−0.014 (3)	−0.002 (3)
C14	0.040 (3)	0.058 (3)	0.052 (3)	0.007 (2)	−0.004 (2)	0.002 (3)
C15	0.051 (3)	0.052 (3)	0.034 (3)	0.011 (2)	−0.006 (2)	−0.008 (2)
C16	0.040 (3)	0.049 (3)	0.042 (3)	0.007 (2)	−0.002 (2)	−0.007 (2)
C17	0.042 (3)	0.053 (3)	0.029 (3)	0.007 (2)	0.003 (2)	−0.001 (2)
C18	0.045 (3)	0.057 (3)	0.046 (3)	0.009 (2)	0.006 (2)	−0.001 (2)
C19	0.063 (4)	0.052 (3)	0.061 (4)	0.006 (3)	0.003 (3)	−0.002 (3)
C20	0.066 (4)	0.062 (4)	0.068 (4)	0.022 (3)	0.005 (3)	0.007 (3)
C21	0.048 (3)	0.087 (5)	0.079 (5)	0.020 (3)	−0.001 (3)	0.008 (4)
C22	0.049 (3)	0.062 (4)	0.066 (4)	0.005 (3)	−0.002 (3)	0.003 (3)
C23	0.048 (3)	0.051 (3)	0.039 (3)	0.013 (2)	0.012 (2)	0.006 (2)
C24	0.068 (4)	0.070 (4)	0.045 (3)	0.009 (3)	0.014 (3)	0.007 (3)
C25	0.083 (5)	0.097 (5)	0.058 (4)	0.020 (4)	0.032 (3)	0.018 (4)
C26	0.075 (5)	0.084 (5)	0.091 (5)	0.007 (4)	0.043 (4)	0.032 (4)
C27	0.065 (4)	0.069 (4)	0.095 (5)	−0.005 (3)	0.025 (4)	0.010 (4)
C28	0.056 (3)	0.061 (3)	0.051 (3)	−0.001 (3)	0.017 (3)	0.005 (3)
C29	0.038 (3)	0.052 (3)	0.049 (3)	0.006 (2)	0.000 (2)	0.001 (2)
C30	0.070 (4)	0.074 (4)	0.086 (5)	−0.009 (3)	0.030 (4)	−0.008 (4)
C31	0.105 (6)	0.086 (5)	0.117 (7)	−0.027 (4)	0.046 (5)	−0.007 (5)
C32	0.094 (5)	0.106 (6)	0.097 (6)	−0.017 (5)	0.049 (4)	0.000 (5)
C33	0.076 (4)	0.107 (6)	0.072 (5)	−0.001 (4)	0.027 (4)	−0.006 (4)
C34	0.054 (3)	0.075 (4)	0.051 (3)	−0.004 (3)	0.007 (3)	−0.005 (3)
C35	0.042 (3)	0.047 (3)	0.037 (3)	0.007 (2)	−0.006 (2)	−0.007 (2)
C36	0.050 (3)	0.060 (3)	0.053 (3)	0.002 (3)	−0.011 (3)	0.003 (3)
C37	0.075 (4)	0.064 (4)	0.071 (4)	−0.004 (3)	−0.020 (3)	0.015 (3)
C38	0.075 (4)	0.069 (4)	0.069 (4)	0.019 (3)	−0.026 (3)	0.009 (3)
C39	0.048 (3)	0.077 (4)	0.068 (4)	0.018 (3)	−0.015 (3)	0.003 (3)
C40	0.044 (3)	0.062 (3)	0.052 (3)	0.006 (3)	−0.007 (2)	−0.001 (3)
C41	0.166 (12)	0.229 (14)	0.204 (16)	−0.004 (10)	−0.006 (10)	0.052 (12)

### Geometric parameters (Å, °)

**Table:**

Cu1—C1	1.951 (6)	C17—C22	1.387 (6)
Cu1—P1	2.2832 (12)	C18—C19	1.388 (6)
Cu1—P3	2.3041 (13)	C18—H18	0.9300
Cu1—P2	2.3291 (12)	C19—C20	1.355 (7)
N1—C1	1.146 (6)	C19—H19	0.9300
O1—C41	1.358 (12)	C20—C21	1.370 (8)
O1—H1	0.8200	C20—H20	0.9300
P1—C3	1.818 (4)	C21—C22	1.383 (7)
P1—C9	1.832 (4)	C21—H21	0.9300
P1—C2	1.840 (4)	C22—H22	0.9300
P2—C17	1.832 (5)	C23—C28	1.370 (7)
P2—C23	1.833 (5)	C23—C24	1.372 (7)
P2—C15	1.846 (5)	C24—C25	1.375 (8)
P3—C29	1.818 (5)	C24—H24	0.9300
P3—C16	1.835 (5)	C25—C26	1.348 (9)
P3—C35	1.835 (5)	C25—H25	0.9300
C2—C2^i^	1.532 (8)	C26—C27	1.363 (9)
C2—H2A	0.9700	C26—H26	0.9300
C2—H2B	0.9700	C27—C28	1.387 (7)
C3—C8	1.382 (6)	C27—H27	0.9300
C3—C4	1.386 (6)	C28—H28	0.9300
C4—C5	1.384 (7)	C29—C30	1.384 (7)
C4—H4	0.9300	C29—C34	1.386 (7)
C5—C6	1.371 (7)	C30—C31	1.391 (8)
C5—H5	0.9300	C30—H30	0.9300
C6—C7	1.378 (7)	C31—C32	1.360 (9)
C6—H6	0.9300	C31—H31	0.9300
C7—C8	1.375 (7)	C32—C33	1.358 (9)
C7—H7	0.9300	C32—H32	0.9300
C8—H8	0.9300	C33—C34	1.377 (8)
C9—C10	1.372 (6)	C33—H33	0.9300
C9—C14	1.381 (6)	C34—H34	0.9300
C10—C11	1.394 (7)	C35—C36	1.374 (7)
C10—H10	0.9300	C35—C40	1.396 (6)
C11—C12	1.352 (8)	C36—C37	1.387 (7)
C11—H11	0.9300	C36—H36	0.9300
C12—C13	1.368 (8)	C37—C38	1.376 (8)
C12—H12	0.9300	C37—H37	0.9300
C13—C14	1.387 (6)	C38—C39	1.355 (8)
C13—H13	0.9300	C38—H38	0.9300
C14—H14	0.9300	C39—C40	1.393 (7)
C15—C16	1.529 (6)	C39—H39	0.9300
C15—H15A	0.9700	C40—H40	0.9300
C15—H15B	0.9700	C41—H41A	0.9600
C16—H16A	0.9700	C41—H41B	0.9600
C16—H16B	0.9700	C41—H41C	0.9600
C17—C18	1.381 (6)		
			
C1—Cu1—P1	107.59 (14)	H16A—C16—H16B	108.2
C1—Cu1—P3	119.11 (14)	C18—C17—C22	117.1 (4)
P1—Cu1—P3	113.60 (5)	C18—C17—P2	123.1 (4)
C1—Cu1—P2	111.79 (14)	C22—C17—P2	119.7 (4)
P1—Cu1—P2	115.07 (5)	C17—C18—C19	121.2 (5)
P3—Cu1—P2	89.03 (4)	C17—C18—H18	119.4
C41—O1—H1	109.5	C19—C18—H18	119.4
C3—P1—C9	103.88 (19)	C20—C19—C18	120.4 (5)
C3—P1—C2	104.93 (19)	C20—C19—H19	119.8
C9—P1—C2	99.06 (18)	C18—C19—H19	119.8
C3—P1—Cu1	117.12 (14)	C19—C20—C21	119.9 (5)
C9—P1—Cu1	114.28 (15)	C19—C20—H20	120.1
C2—P1—Cu1	115.39 (14)	C21—C20—H20	120.1
C17—P2—C23	99.5 (2)	C20—C21—C22	119.9 (5)
C17—P2—C15	103.7 (2)	C20—C21—H21	120.1
C23—P2—C15	104.3 (2)	C22—C21—H21	120.1
C17—P2—Cu1	125.84 (15)	C21—C22—C17	121.5 (5)
C23—P2—Cu1	118.46 (16)	C21—C22—H22	119.3
C15—P2—Cu1	102.56 (15)	C17—C22—H22	119.3
C29—P3—C16	105.0 (2)	C28—C23—C24	118.8 (5)
C29—P3—C35	102.3 (2)	C28—C23—P2	118.9 (4)
C16—P3—C35	101.2 (2)	C24—C23—P2	122.2 (4)
C29—P3—Cu1	123.19 (17)	C23—C24—C25	120.7 (6)
C16—P3—Cu1	103.70 (15)	C23—C24—H24	119.7
C35—P3—Cu1	118.66 (16)	C25—C24—H24	119.7
N1—C1—Cu1	176.6 (5)	C26—C25—C24	120.1 (6)
C2^i^—C2—P1	113.6 (4)	C26—C25—H25	120.0
C2^i^—C2—H2A	108.8	C24—C25—H25	120.0
P1—C2—H2A	108.8	C25—C26—C27	120.6 (6)
C2^i^—C2—H2B	108.8	C25—C26—H26	119.7
P1—C2—H2B	108.8	C27—C26—H26	119.7
H2A—C2—H2B	107.7	C26—C27—C28	119.5 (6)
C8—C3—C4	117.7 (4)	C26—C27—H27	120.2
C8—C3—P1	124.8 (3)	C28—C27—H27	120.2
C4—C3—P1	117.5 (3)	C23—C28—C27	120.4 (6)
C5—C4—C3	121.2 (5)	C23—C28—H28	119.8
C5—C4—H4	119.4	C27—C28—H28	119.8
C3—C4—H4	119.4	C30—C29—C34	118.9 (5)
C6—C5—C4	119.9 (5)	C30—C29—P3	123.5 (4)
C6—C5—H5	120.1	C34—C29—P3	117.6 (4)
C4—C5—H5	120.1	C29—C30—C31	119.3 (6)
C5—C6—C7	119.8 (5)	C29—C30—H30	120.3
C5—C6—H6	120.1	C31—C30—H30	120.3
C7—C6—H6	120.1	C32—C31—C30	120.7 (7)
C8—C7—C6	119.9 (5)	C32—C31—H31	119.7
C8—C7—H7	120.1	C30—C31—H31	119.7
C6—C7—H7	120.1	C33—C32—C31	120.5 (6)
C7—C8—C3	121.5 (5)	C33—C32—H32	119.7
C7—C8—H8	119.2	C31—C32—H32	119.7
C3—C8—H8	119.2	C32—C33—C34	119.8 (6)
C10—C9—C14	118.2 (4)	C32—C33—H33	120.1
C10—C9—P1	118.9 (4)	C34—C33—H33	120.1
C14—C9—P1	122.8 (3)	C33—C34—C29	120.8 (6)
C9—C10—C11	120.6 (5)	C33—C34—H34	119.6
C9—C10—H10	119.7	C29—C34—H34	119.6
C11—C10—H10	119.7	C36—C35—C40	118.9 (4)
C12—C11—C10	120.4 (5)	C36—C35—P3	119.1 (4)
C12—C11—H11	119.8	C40—C35—P3	122.0 (4)
C10—C11—H11	119.8	C35—C36—C37	120.6 (5)
C11—C12—C13	120.1 (5)	C35—C36—H36	119.7
C11—C12—H12	120.0	C37—C36—H36	119.7
C13—C12—H12	120.0	C38—C37—C36	119.9 (6)
C12—C13—C14	119.7 (5)	C38—C37—H37	120.0
C12—C13—H13	120.1	C36—C37—H37	120.0
C14—C13—H13	120.1	C39—C38—C37	120.3 (5)
C9—C14—C13	121.0 (5)	C39—C38—H38	119.9
C9—C14—H14	119.5	C37—C38—H38	119.9
C13—C14—H14	119.5	C38—C39—C40	120.6 (5)
C16—C15—P2	112.0 (3)	C38—C39—H39	119.7
C16—C15—H15A	109.2	C40—C39—H39	119.7
P2—C15—H15A	109.2	C39—C40—C35	119.7 (5)
C16—C15—H15B	109.2	C39—C40—H40	120.2
P2—C15—H15B	109.2	C35—C40—H40	120.2
H15A—C15—H15B	107.9	O1—C41—H41A	109.5
C15—C16—P3	109.8 (3)	O1—C41—H41B	109.5
C15—C16—H16A	109.7	H41A—C41—H41B	109.5
P3—C16—H16A	109.7	O1—C41—H41C	109.5
C15—C16—H16B	109.7	H41A—C41—H41C	109.5
P3—C16—H16B	109.7	H41B—C41—H41C	109.5
			
C1—Cu1—P1—C3	160.9 (2)	P2—C15—C16—P3	54.5 (4)
P3—Cu1—P1—C3	26.84 (16)	C29—P3—C16—C15	−172.6 (3)
P2—Cu1—P1—C3	−73.77 (16)	C35—P3—C16—C15	81.3 (3)
C1—Cu1—P1—C9	−77.3 (2)	Cu1—P3—C16—C15	−42.1 (3)
P3—Cu1—P1—C9	148.63 (15)	C23—P2—C17—C18	−135.9 (4)
P2—Cu1—P1—C9	48.02 (16)	C15—P2—C17—C18	−28.6 (4)
C1—Cu1—P1—C2	36.6 (2)	Cu1—P2—C17—C18	88.2 (4)
P3—Cu1—P1—C2	−97.46 (15)	C23—P2—C17—C22	45.0 (4)
P2—Cu1—P1—C2	161.93 (15)	C15—P2—C17—C22	152.3 (4)
C1—Cu1—P2—C17	130.6 (2)	Cu1—P2—C17—C22	−90.8 (4)
P1—Cu1—P2—C17	7.5 (2)	C22—C17—C18—C19	−0.9 (7)
P3—Cu1—P2—C17	−108.3 (2)	P2—C17—C18—C19	−179.9 (4)
C1—Cu1—P2—C23	2.0 (2)	C17—C18—C19—C20	0.2 (8)
P1—Cu1—P2—C23	−121.14 (18)	C18—C19—C20—C21	0.1 (9)
P3—Cu1—P2—C23	123.13 (18)	C19—C20—C21—C22	0.3 (9)
C1—Cu1—P2—C15	−112.1 (2)	C20—C21—C22—C17	−1.1 (9)
P1—Cu1—P2—C15	124.81 (16)	C18—C17—C22—C21	1.3 (8)
P3—Cu1—P2—C15	9.07 (16)	P2—C17—C22—C21	−179.6 (4)
C1—Cu1—P3—C29	−112.1 (2)	C17—P2—C23—C28	−112.0 (4)
P1—Cu1—P3—C29	16.2 (2)	C15—P2—C23—C28	141.1 (4)
P2—Cu1—P3—C29	133.3 (2)	Cu1—P2—C23—C28	28.0 (4)
C1—Cu1—P3—C16	129.4 (2)	C17—P2—C23—C24	63.0 (4)
P1—Cu1—P3—C16	−102.25 (17)	C15—P2—C23—C24	−43.9 (5)
P2—Cu1—P3—C16	14.82 (17)	Cu1—P2—C23—C24	−157.0 (4)
C1—Cu1—P3—C35	18.2 (2)	C28—C23—C24—C25	−0.8 (8)
P1—Cu1—P3—C35	146.56 (17)	P2—C23—C24—C25	−175.8 (4)
P2—Cu1—P3—C35	−96.37 (17)	C23—C24—C25—C26	1.7 (9)
C3—P1—C2—C2^i^	−72.9 (4)	C24—C25—C26—C27	−1.4 (10)
C9—P1—C2—C2^i^	−180.0 (4)	C25—C26—C27—C28	0.3 (10)
Cu1—P1—C2—C2^i^	57.6 (4)	C24—C23—C28—C27	−0.3 (8)
C9—P1—C3—C8	92.8 (4)	P2—C23—C28—C27	174.8 (4)
C2—P1—C3—C8	−10.7 (4)	C26—C27—C28—C23	0.6 (9)
Cu1—P1—C3—C8	−140.2 (3)	C16—P3—C29—C30	−0.7 (5)
C9—P1—C3—C4	−87.4 (4)	C35—P3—C29—C30	104.6 (5)
C2—P1—C3—C4	169.1 (3)	Cu1—P3—C29—C30	−118.6 (5)
Cu1—P1—C3—C4	39.7 (4)	C16—P3—C29—C34	−178.7 (4)
C8—C3—C4—C5	−0.2 (7)	C35—P3—C29—C34	−73.4 (4)
P1—C3—C4—C5	179.9 (4)	Cu1—P3—C29—C34	63.4 (4)
C3—C4—C5—C6	−0.1 (8)	C34—C29—C30—C31	0.3 (9)
C4—C5—C6—C7	0.9 (8)	P3—C29—C30—C31	−177.6 (5)
C5—C6—C7—C8	−1.3 (8)	C29—C30—C31—C32	−1.3 (12)
C6—C7—C8—C3	1.0 (8)	C30—C31—C32—C33	1.7 (13)
C4—C3—C8—C7	−0.2 (7)	C31—C32—C33—C34	−1.0 (12)
P1—C3—C8—C7	179.6 (4)	C32—C33—C34—C29	0.0 (10)
C3—P1—C9—C10	151.4 (4)	C30—C29—C34—C33	0.3 (9)
C2—P1—C9—C10	−100.6 (4)	P3—C29—C34—C33	178.4 (5)
Cu1—P1—C9—C10	22.6 (4)	C29—P3—C35—C36	159.4 (4)
C3—P1—C9—C14	−33.0 (4)	C16—P3—C35—C36	−92.4 (4)
C2—P1—C9—C14	74.9 (4)	Cu1—P3—C35—C36	20.2 (4)
Cu1—P1—C9—C14	−161.8 (3)	C29—P3—C35—C40	−22.0 (4)
C14—C9—C10—C11	0.0 (8)	C16—P3—C35—C40	86.2 (4)
P1—C9—C10—C11	175.7 (4)	Cu1—P3—C35—C40	−161.3 (3)
C9—C10—C11—C12	0.6 (9)	C40—C35—C36—C37	−0.3 (8)
C10—C11—C12—C13	−1.1 (9)	P3—C35—C36—C37	178.3 (4)
C11—C12—C13—C14	1.0 (9)	C35—C36—C37—C38	1.0 (9)
C10—C9—C14—C13	−0.1 (7)	C36—C37—C38—C39	−1.1 (9)
P1—C9—C14—C13	−175.7 (4)	C37—C38—C39—C40	0.6 (9)
C12—C13—C14—C9	−0.4 (8)	C38—C39—C40—C35	0.1 (8)
C17—P2—C15—C16	93.9 (3)	C36—C35—C40—C39	−0.2 (7)
C23—P2—C15—C16	−162.3 (3)	P3—C35—C40—C39	−178.8 (4)
Cu1—P2—C15—C16	−38.3 (3)		

Symmetry codes: (i) −*x*+1/2, −*y*+1/2, −*z*+1.

### Hydrogen-bond geometry (Å, °)

**Table:**

*D*—H···*A*	*D*—H	H···*A*	*D*···*A*	*D*—H···*A*
O1—H1···N1^i^	0.82	2.02	2.829 (11)	171

Symmetry codes: (i) −*x*+1/2, −*y*+1/2, −*z*+1.
